# Female horses spontaneously identify a photograph of their keeper, last seen six months previously

**DOI:** 10.1038/s41598-020-62940-w

**Published:** 2020-04-14

**Authors:** Léa Lansade, Violaine Colson, Céline Parias, Miléna Trösch, Fabrice Reigner, Ludovic Calandreau

**Affiliations:** 1 PRC, INRAE, CNRS, IFCE, University Tours, F-37380 Nouzilly, France; 2 INRAE, UR1037 Fish Physiology and Genomics, F-35000 Rennes, France; 3 UEPAO, F-37380 Nouzilly, France

**Keywords:** Zoology, Animal behaviour

## Abstract

Horses are capable of identifying individual conspecifics based on olfactory, auditory or visual cues. However, this raises the questions of their ability to recognize human beings and on the basis of what cues. This study investigated whether horses could differentiate between a familiar and unfamiliar human from photographs of faces. Eleven horses were trained on a discrimination task using a computer-controlled screen, on which two photographs were presented simultaneously (32 trials/session): touching one was rewarded (S+) and the other not (S−). In the training phase, the S+ faces were of four unfamiliar people which gradually became familiar over the trials. The S− faces were novel for each trial. After the training phase, the faces of the horses’ keepers were presented opposite novel faces to test whether the horses could identify the former spontaneously. A reward was given whichever face was touched to avoid any possible learning effect. Horses touched the faces of keepers significantly more than chance, whether it was their current keeper or one they had not seen for six months (t = 3.65; p < 0.004 and t = 6.24; p < 0.0001). Overall, these results show that horses have advanced human face-recognition abilities and a long-term memory of those human faces.

## Introduction

Individual recognition is essential for social species, it enables animals to differentiate between individuals and to adapt their behavior accordingly (for reviews see^1,2^). This can be based on different sensory signals (odor, audition or vision), which are indeed often processed in a combined non-exclusive way^3^. Among visual information that has been processed, facial recognition has certainly been studied the most. In general, studies have investigated recognition of faces of the same species as that being tested. For example, capuchin monkeys^4^ or macaques^5^ are able to recognize the faces of members of their group. It is also true for sheep, cattle and pigeons which are able to learn to discriminate between individual faces of their conspecifics in photographs, even when the individual was not known in real life^6–8^.

In certain species, particularly domestic species which share a long evolutionary history with human beings, studies are beginning to investigate not only recognition of conspecifics but also of human faces. The tasks completed in these studies varied in their difficulty, from pigs simply differentiating between a human face and the back of the head^9^, to dogs or sheep recognizing the face of a person known in real life from an image^10–12^.

For both inter and intra specific recognition, real people^10^ or photographs^7,9,12^ have been used. The advantage of the latter is that it excludes any risk of interference from other sensory information, particularly olfactory cues. However, using photographs can be particularly complex for non-human species^13^. Indeed, the animal must understand that a relationship exists between the real individual and his/her 2-dimentional image seen in a photograph. Moreover, in a photograph much visual information is lost, such as depth, perspective or movement^14^. The colors or size of the original can also be modified. While primates, such as capuchins or macaques are able to process this information and recognize photographs of their conspecifics^4,5^, this task is more difficult for species such as the dog. Dogs can easily recognize faces of familiar people in real life^10^, but find it more difficult when shown only a photograph, and in particular when only the center of the face is visible^12^. Surprisingly, a recent study has shown that sheep can recognize their keeper from a photograph alone^11^. However, the authors concluded that these results needed to be interpreted with care because they could have been due to a simple learning effect occurring during the test.

To date, no study has demonstrated that horses are able to recognize a human known in real life on the basis of a photograph of that person’s face alone. However, some indications suggest that it is within their abilities. Firstly, it has been demonstrated that they are capable of cross-modal recognition (associating sight and corresponding neighing or voice) not only of other horses^15^ but also of humans. When they see a familiar person but hear the voice of someone else (an incongruent situation), they look longer in the direction of the auditory cue than when the person and the voice are congruent^16^. Horses are also capable of detecting emotional expressions on the faces of conspecifics^17^ and also of humans including in photographs^18–20^. For example in this last study^19^ horses which had been presented with a photograph of a person who was either angry or happy adapted their behavior when they were in front of that person in real life. They had a left gaze bias and spent more time engaging in displacement behaviors (e.g. scratching, floor sniffing) when viewing people who had been angry in photographs. Secondly, in a study in 2010, Stone demonstrated that four horses were able to learn to discriminate between photographs of different people and then transfer that facial recognition in a field test by spending more time with the person whose photograph had previously been associated with a reward^21^. These studies suggest that horses could possess skills to perceive and integrate cues contained in photographs of faces, and could use them to recognize humans. However, all the studies on horses mentioned using photographs involved transferring information seen in a photograph to a person in real life. To date, no study has investigated the opposite, that is to say transferring from a real life person to his/her photograph, and more particularly whether a horse is capable of recognizing a person known in real life from a photograph of their face.

Moreover, to be able to identify people it is necessary for social species to memorize information and to recall it at a later date. In humans, the memory of faces can be remarkable as we can recognize faces of people we have not seen for over 50 years^22^. However, the degree to which horses are capable of remembering the identity of people they have spent time with is unknown. Considering the life-span of a horse of between 25 and 30 years, and particularly regarding previous studies on the horse’s ability to memorize information, it would be reasonable to think that a horse could remember information for a period of several months. Indeed, it can correctly recall the conditioning procedure several years later^23,24^. In addition, horses can also remember the type of relationship (positive or negative) they had experienced with a human several months after the interaction^25,26^. However, no information is available about a horse’s potential capacity to remember faces over time.

The aims of this study were thus to determine whether horses were able to spontaneously differentiate a photograph of an unfamiliar face from: 1) the face of a person they were regularly in contact with in real life, but which they had never seen in a photograph; 2) the photograph of the face of a person they had not seen for six months.

To that end, we conducted an experiment in two stages based on computer-controlled screen testing. This protocol was based on that used by Knolle *et al*. on sheep^11^, with certain points adapted. The complete method is described at the end of the manuscript. Briefly, we taught 11 horses to touch a photograph of a “recurrent” face on a screen when it was presented opposite a systematically different novel face. In this first stage, the recurrent faces of four people unfamiliar to the horse in real life were presented on the screen, and these gradually became familiar over successive trials (32 per session). Several intermediary steps were conducted to reach this objective (initially, one of the four faces was presented opposite a black circle, and then opposite novel objects and finally opposite novel faces). Once horses had learned to touch the recurrent face during the training phase, they performed probe tests in which they had to choose between the face of a person familiar in real life (but until then never seen in a photograph) and a novel face (32 different novel faces were presented in each session). At this stage to ensure the results could not be due to new learning and were due to a spontaneous choice, the horse was rewarded regardless of whether it touched the familiar or the unfamiliar face. Finally, using the same procedure we tested whether the horses could identify the face of a person that they had not seen for six months.

We hypothesized that in the probe tests the horses would touch the photograph of the familiar person significantly more frequently than chance level. We also conducted a control test at the end of the experiment to check that the horses had not based their responses on clues other than familiarity with the person in real life to identify the familiar face. Instead of the keeper’s face, we used the face of an unfamiliar person. We thus hypothesized that the horses would not touch this control face more frequently than chance.

## Results

### Pre-training

After a lengthy familiarization process to human and to the test arena, horses were familiarized with the choice test on the screen in four sessions (the week immediately preceding the training), during which they had to learn to touch an image of a horse’s head rather than a black circle (see Method). The performance (percentage of correct responses out of the 32 trials conducted) increased significantly for all the horses (Friedman test: Q = 28.37; P < 0.0001), and at the end of the four sessions, they had all responded correctly in more than 75% of the trials.

### Training

**Figure 1:**
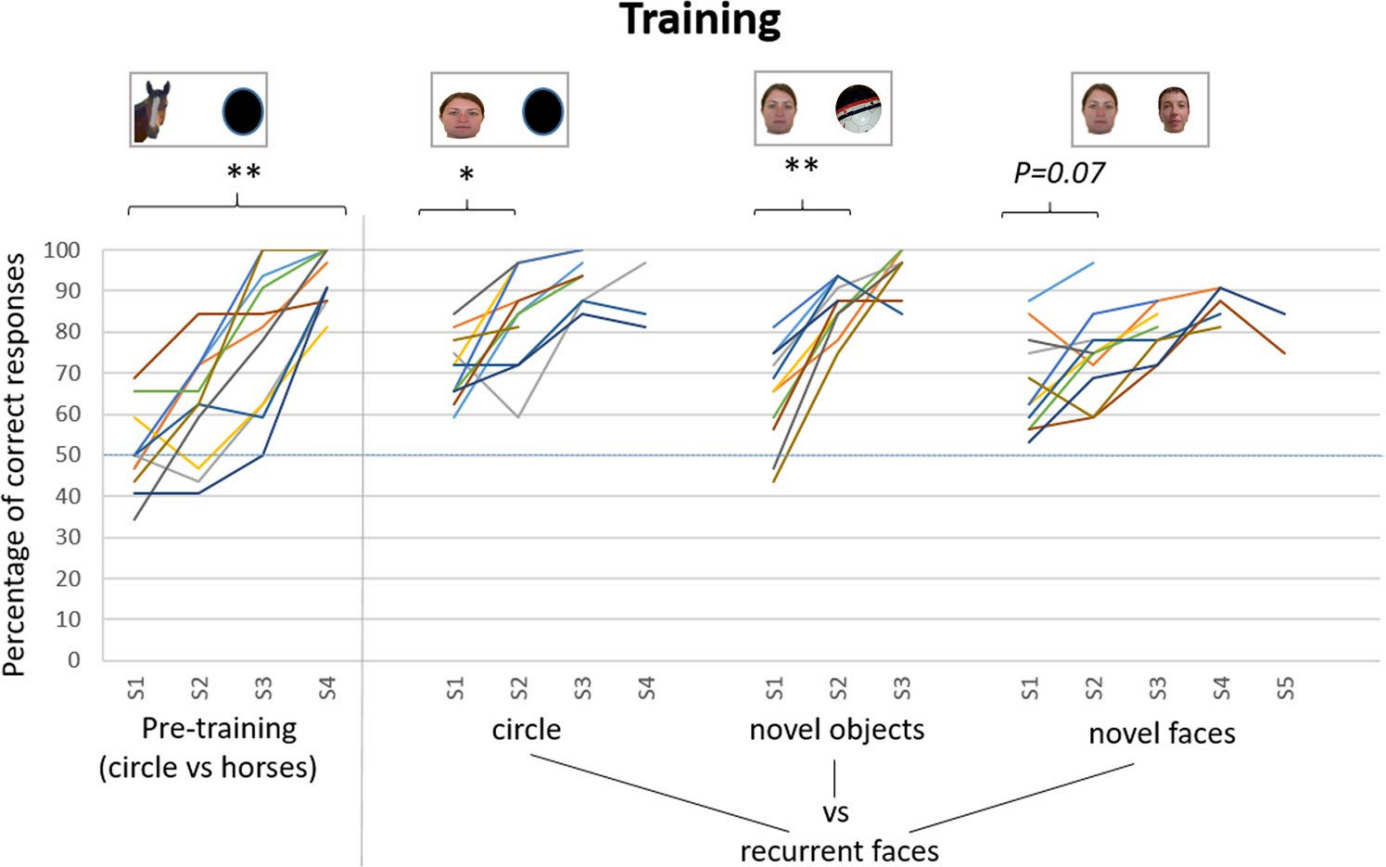
Individual learning curves for the different training stages. S1 to S5: session 1 to 5 (32 trials per session). :  Recurrent faces (the same four different faces were always used). : Novel objects (different for each trial). : Novel faces (different for each trial). The statistical values indicated correspond to comparisons of the percentage of correct responses between sessions, when carried out by all the subjects (N = 11): Friedman test for the pre-training or Wilcoxon tests for the other stages of training. **P < 0.01; *P < 0.05.

The horses were then trained to touch one of the four recurrent human faces rather than the black circle. Their performance improved significantly from the second session (Wilcoxon test: V = 95.62; P = 0.021, Fig. 1). We could only compare the percentage of correct responses between the first two training sessions because only these two sessions involved all the horses, as once the horses reached a 75% success rate on two consecutive sessions they moved on to the next stage. The subjects reached this criterion within two to four sessions (Table 1).

**Table 1:** Number of horses reaching the success criterion according to the number of sessions.

		S2	S3	S4	S5
*Training stage*	Recurrent faces *vs*. black circle	3	5	3	
	Recurrent faces *vs*. novel objects	3	8		
	Recurrent faces *vs*. novel faces	3	3	3	2

Success criterion: 75% of correct responses on two consecutive sessions; S2 to S5: session 2 to 5 (32 trials per session).

After reaching this criterion, the horses were trained to touch one of the four recurrent faces rather than an image of a novel object (an image of a different novel object was used for each trial within a session, with a new series of 32 images used for each session). Here the performance also increased significantly between the first and the second session (Wilcoxon test: V = 125.25; P = 0.003, subsequently not tested since some animals had then moved on to the next stage, Fig. 1). The horses reached the success criterion of a 75% success rate on two consecutive sessions in the second or third session (Table 1).

Finally, the horses were trained to touch the photograph of one of the four recurrent faces rather than a novel face (images of 32 different novel faces per session, with a new series for each session). Performance tended to increase from the second session (Wilcoxon test: V = 125.62; P = 0.07, subsequently not tested, Fig. 1). The horses reached the success criterion within two to five sessions (Table 1).

### Probe tests

**Figure 2:**
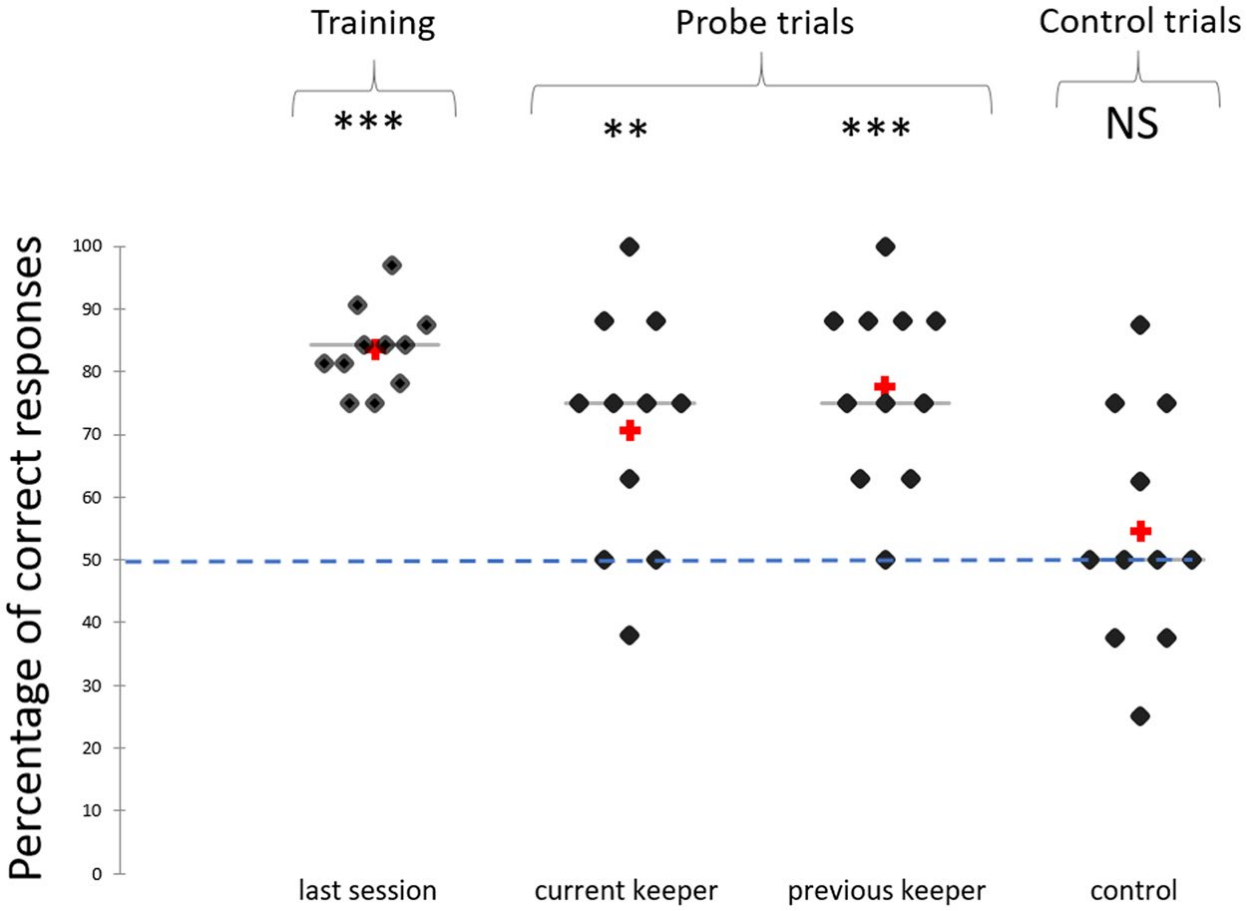
Percentage of correct responses during training, probe and control trials. The last training session (32 trials) corresponded to the last time the animal was trained to touch the recurrent faces rather than a novel face (according to the individual performance, this session was not the same for all subjects). During the probe and control trials (8 trials each) rewards were given whichever face was touched. NS: Non-significant; **P < 0.01; ***P < 0.001, t-test, calculated according to the level of chance (50%), N = 11. : mean, : median.

During the probe trials with the current keeper, horses had to choose between the face of their current keeper and a novel face (8 different probe trials, interspersed during the session, see Method). Horses’ performances during the probe trials were significantly above chance (t = 3.65; P < 0.004, Cohen’s d = 1.56, Fig. 2). For the probe trials with the previous keeper, horses had to choose between the face of the keeper they had not seen for 6 months and a novel face. Their performances were again significantly above chance (t = 6.24; P < 0.0001, Cohen’s d = 2.66, Fig. 2).

### Control test

A control test was conducted at the end of the experiment to check whether the horses had not used another criterion than familiarity of the person in real life to choose the correct face during the probe trials. This consisted in repeating the same procedure as the probe tests (8 probe trials, interspersed during the session), but instead of the keeper’s face, the face of the same unfamiliar person was presented. The results show that the performances were not significantly above chance for the control face (t = 0.8; P = 0.44, Cohen’s d = 0.34, Fig. 2).

### Side bias during the probe and control trials

There was no preference of side during the two types of probe trials (current keeper, t = 0.89, P = 0.40; Cohen’s d = 0.37, previous keeper: t = 0, P = 1, Cohen’s d = 0). Nevertheless, the horses had a general preference for the right side of the screen for the control trials (t = 2.85, P = 0.017, Cohen’s d = 1.21, Supplementary Fig. S1).

## Discussion

In line with our hypotheses, the results show that having learned to touch a recurrent face during the training sessions, the horses preferentially chose the face of their current or previous keeper instead of a novel face during the probe trials. These results can be explained in different ways: the horses could have identified the familiarity of the person in real life, or they could have based their choice on clues that had no connection with recognizing familiarity (experimental bias). The following section discusses these biases.

One experimental bias could have been linked to the quality of the photographs used. Despite all the care taken in processing the photographs (all were checked regarding contrast and brightness), there could have been more similarities between the photographs of the recurrent and the keepers faces than with the novel faces that were used. The horses could have detected this similarity, which in theory could have enabled them to identify these photographs. For this reason we conducted a control test at the end of the experiment, in which we used a photograph of an unfamiliar person taken under exactly the same conditions and presented in the same way as the familiar faces. The results showed that performance in the control test did not differ from a chance level. Bias based on photograph quality is thus unlikely. A second experimental bias could have been due to the fact that a same photograph reappeared eight times over a session, which would have enabled the horses to identify it rapidly. This bias was identified and discussed by Knolle *et al*.^11^ in their study on sheep. However, in contrast to their study, during our probe trials a reward was given whether the horse touched the familiar or unfamiliar face, as is generally required for this type of protocol (e.g.^4^). In theory, this avoids animals learning to touch one particular face as the trial progresses. Moreover, during the control tests we presented the same photograph of an unfamiliar face eight times throughout the session, following the same procedure as for the keepers’ faces. During the control trials, the horses’ performances were not significantly different to a chance level, indicating that they could not have identified the photograph simply because it had appeared repeatedly. This second bias can thus be rejected.

In view of the performances recorded and having rejected the alternative explanations developed above, this study implies three main results regarding the cognitive abilities of horses. Firstly, our results suggest that horses are able to spontaneously understand the representational nature of 2-dimensional images. It has already been demonstrated that horses can transfer information perceived in two dimensions (an image) to 3-dimensional information (a real person)^19,21^. In our experiment, we showed that horses were also capable of the opposite process: transferring 3-dimensional information (real people) to 2-dimensional information (photographs). It is not apparent that animals would have this ability^13^ because photographs represent only a small part of the information concerning reality: no recognition is possible through odors, behavior or voice, for example, and there is also a loss of visual information such as depth, perspective or movement^14^. Thus, some species, such as dogs, have difficulties recognizing photographs of faces and particularly when only the central part is visible^12^, whereas they are quite capable of recognizing faces in real life conditions^10^. This does not appear to be the case for horses, although to confirm this requires testing the two species using exactly the same experimental paradigm. Moreover, it has been reported that horses have weak visual acuity compared to human beings^27^, and a blind spot in front of their head. This would thus imply difficulties in perceiving enough details of a face on a screen for the horse to be able to identify a photograph of their keeper, but this was not observed here. It is possible that by moving their heads slightly the horses managed to avoid their blind spot. Finally, it is important to note here that the horses in our study had not previously been trained to link real objects and their 2-D images. Indeed, research has suggested that this link might need to be learned, being the case even in humans^28^. The horses in our study did not appear to have any difficulties adjusting between a real person and a photograph of their face, without prior learning.

Secondly, this shows that the horses had a good ability to identify faces. During the training, they first learned to identify unfamiliar human faces through conditioning with images being presented repeatedly, thus confirming findings previously highlighted in domestic species such as dogs^12^ and sheep^11^. This also partially confirms Stone’s study in horses, which demonstrated that they were able to identify a face, in particular during conditioning^21^. However, Stone’s study involved the same two pairs of faces which were presented with a reward given for only one of them. In our study, the degree of complexity was probably greater as there were four different faces to recognize, presented opposite a large number of novel faces. During the probe tests, the horses were thus able to apply the rule ‘touch the recurrent face’ to ‘touch a face known in real life’ to identify their keeper’s face. It has previously been demonstrated that several species, including domestic ungulates, could recognize individual conspecifics from photographs (capuchins^4^, sheep^6^ and cattle^7^). Cross-modal recognition of conspecifics also exists in horses; they are able to associate a neigh with the image of a specific horse^15^. However, few studies on recognizing human faces known in real life have been conducted on domestic animals. In sheep^11^, findings suggest that they also have this ability, but as discussed above the authors proposed alternative explanations for their results. The present study is the first time to our knowledge that horses’ capacity to recognize the photograph of the face of a person known in real life has been demonstrated without prior learning. The fact that horses have been domesticated for thousands of years^29^, not just for their meat or milk, but to interact specifically with humans for work (to draw loads or carry a rider) could explain why this skill might have been selected over successive generations and may be more developed in modern-day domestic horses. Unfortunately, since wild horses have totally disappeared^30^, we will never be able to test this hypothesis formally. It still remains possible to test this hypothesis, by comparing modern-day domestic horses and groups of feral horses (e.g. Przewalski horses) that have not undergone selection for their interaction with humans for many generations. Moreover, it would be interesting to investigate whether this inter-species recognition skill is specific to humans or whether it exists for other species horses come in contact with in everyday life (sheep, dogs, cattle, etc.).

Finally, probably the most noteworthy finding of this study is the information provided on horses’ long-term memory capacities. The fact that the horses recognized the photograph of a person they had not seen for six months shows that they have a good memory for faces, a fact that was unknown until now. Regarding long-term memory in horses, previous studies have shown that horses can remember operant tasks learned approximately two years previously, with no decrease in performance^24^. Another study, conducted on only three horses showed that they recalled correctly complex problem-solving strategies seven years later^23^. Regarding the more specific human-animal relationship, horses could remember interactions they had had with human beings five months^26^, or even a year previously^25^. The present study shows that beyond remembering what they have learned or the interactions they have had with humans, horses also have an excellent memory of people and particularly of their faces.

Lastly, we will discuss two additional points: side bias and gender effects. Regarding side bias, there was no side preference during the probe trials with familiar keepers. However, there was an effect in the control test when the horses did not manage to solve the task. This side bias was probably only a symptom of the animal’s failure: when it did not understand the rule, it chose a simple rule: always go to the same side. This effect is typically observed in this type of protocol, for example, see^12^. In our study, the horses preferred to go to the right side. This could be linked to a potential hemispheric lateralization. In particular, lateralization effects are reported for emotional processes, but also during learning tasks^31,32^. For example, goats showed a right-hemisphere bias of processing of visuospatial cues in a maze before they had understood the rule^33^. Horses also show right hemisphere preference for social processing^34,35^. That could have explained why in our study, they could have chosen the right side of the screen to observe the faces with their left eye when they were unsure and trying to decide. However, although our test area appeared symmetric, the horses could have noticed signs which we were unaware of leading them to touch the right side of the screen preferentially when they did not know which face to touch.

Regarding gender effects, this study was conducted only on females and the photographs presented were also only of women. Given the number of subjects, we could not multiply the factors. In the future, this study could be extended to test male horses and faces of men. For instance, in dogs, it has been suggested that males could be slightly better than females at recognizing their owner^10^.

This study opens up several other avenues to be investigated. First, it is interesting to note that the keeper in the photographs presented during our probe tests was only involved in positive situations (feeding and training with positive reinforcement). The results might be different if the horses were tested with photographs of a person perceived as aversive. In that case, we might expect avoidance behavior or anxiety. It would be interesting to test this possibility in the future. Second, it would be interesting to investigate the mechanisms underlying face recognition in horses and compare them to those in humans, monkeys and even dogs^36,37^, for whom cortical brain regions have been identified as focusing preferentially on processing faces. This could be achieved non-invasively using fMRI or perhaps more realistically with EEG, an emerging technique in horses^38^. Finally, it could be interesting to replicate this type of experiment using different photographs of recurrent and known people to determine whether horses are able to recognize different portraits of the same person (e.g., front or side profiles, or with different hair styles), and whether certain images were more likely to be chosen correctly.

## Conclusions

In conclusion, these results show that horses have advanced face-recognition abilities, and are able, like humans, to differentiate between a photograph of a familiar and unfamiliar individual, even when the faces did not belong to their own species. Moreover, they have a long-term memory of human faces. In addition, by highlighting that the visual mode of facial recognition from photographs is enough to identify a familiar human improves our understanding of the signals underlying individual recognition in horses. More generally, as discussed by Nawroth *et al*. in their review paper^39^, the degree to which domestic ungulates can demonstrate sophisticated socio-cognitive skills and are sensitive to subtle behavioral cues of conspecifics and humans should be taken into account in our everyday interactions with these animals and raises new ethical issues in relation to how we manage livestock in general.

Finally, from a strictly methodological perspective, this study confirms that horses are capable of interacting with a computer-controlled screen within a conditioning framework, as has previously been demonstrated^40^, and are also able to understand the representational nature of 2-dimensional images. Testing animals in front of a computer screen displaying images of real life seems an interesting tool to test various other cognitive skills in horses in the future.

## Methods

### Ethics statement

This experimental protocol was approved by the Val de Loire Ethics Committee (CEEA VdL, France). Animal care and experimental treatments complied with the French and European guidelines for housing and care of animals used for scientific purposes (European Union Directive 2010/63/EU) and were performed under authorization and supervision of official veterinary services (agreement number F371752 delivered to the UEPAO animal facility by the veterinary service of the Département d’Indre et Loire, France). The animals were not food deprived during the experiment and did not undergo any invasive procedures. They lived in groups and went out to pasture daily. All methods were carried out in accordance with relevant guidelines and regulations for direct human involvement in the study. All the people participating in the study gave their informed consent. Informed consent was also obtained to publish the images of the experimenters in an online open access publication.

### Subjects and relationships with humans

The study was conducted on 11 three-year-old female Welsh breed horses. They were kept in a group at pasture or in a large stall with straw bedding under bad weather conditions. They had free access to fodder and water. These horses were bred and spent their whole life at the French National Institute for Agricultural Research (PAO, INRA, Animal Physiology Experimental Facility, DOI: 10.15454/1.5573896321728955E12). A team of around 15 people shared in the care of the horses from their birth, mucking out the stables, distributing hay and pellets, and handling them (putting on a halter, walking in hand and carrying out basic health care such as clipping hooves, vaccinations, worming, weighing, etc.). In addition, interns also handled the horses during internships varying from one to five months. The horses could also see visitors during the day.

### Testing area

The animals were tested individually in a testing area (6 m × 4 m) equipped with a video camera. A system consisting of a tactile screen (1.02 m × 0.57 m) linked to a computer with an automatic pellet distributor was located at the end of the area. Individual horses were led into the area and let loose in front of the system. For safety reasons, an experimenter remained in the test area, with their back turned to the horse and screen. He continually observed the proceedings on a small video surveillance screen, ready to intervene between trials in the event of a horse becoming distressed or risking injury with the equipment. Importantly, he cannot see which items are displayed on the screen. In that way, he could not influence the horse choice. The experimenter was instructed to remain motionless during the trials. A second experimenter controlled the computer, out of sight of the horses.

### Familiarization with humans and the equipment

For 11 months preceding the study, the horses were handled five days a week for 10 to 20 minutes per day. This was initially to habituate them to being led in a halter and then they were taken alone into the test area to receive food, and finally they learned to touch shapes that appeared on the screen in order to receive a reward. The horses required this long familiarization phase because they remained easily distracted and rapidly lost interested in the screen. After this phase, the horses were able to do a series of trials without becoming distracted.

### General procedure

To condition the horses to touch photographs of familiar faces, we adapted the protocol used by Knolle *et al*.^11^. Each session consisted of 32 consecutive trials. The sessions were conducted between 9:00 and 17:00, five days a week and with a maximum of one session per day. The horse was haltered in its living area and led to the test area where it was let loose. Each trial began with a blank screen. After 30 s, two images appeared simultaneously on the screen: a rewarded (S+) and an unrewarded (S−) image. When the horse touched one of the images with its nose or did nothing for 30 s the screen became blank again. When the horse touched an S+ image, 5 g of pellets fell automatically into a feeding trough located just below the screen. The side on which the S+ image was presented was chosen randomly (the same number of times on the right and left side of the screen and never more than two consecutive times on the same side). A correction procedure was used: when an incorrect choice or no choice was made, the trial was repeated four times or until the horse touched the corrected image, whichever occurred first. Only the result of the first presentation was included in the analysis. If the horse chose wrongly all four times, a new trial began. In the end, no maximum time duration for the trials was necessary since the horses always chose quickly once the photographs appeared on the screen and no animal refused to make a choice during the tests.

### Pre-training

During pre-training, the horses carried out four sessions in which they were simultaneously presented with a photograph of a familiar horse’s head (S+) opposite a black circle (S−). The same photograph was presented throughout the pre-training phase.

### Training phases

During the training phases, S+ was always a photograph of a human face chosen from the same four faces (the “recurrent faces”). The choice of four different faces was based on the protocol used successfully on sheep^11^. The S− image depended on the training stage: for the first training stage it was a black circle, for the second it was a novel object and for the third it was a novel face. “Novel” indicated that the stimulus was new for each trial, thus 32 new objects or new faces were presented during each session. These series of images were different for each session. All the horses were presented with the four “recurrent faces” (S+) during each session and each of these “recurrent faces” was presented eight times during each session in a semi-randomized order (one “recurrent face” was never presented more than twice consecutively). The horse moved on to the next stage once it had reached at least 75% of correct responses over two consecutive sessions.

### Probe tests with current or previous keeper

Over the 32 trials in one session, 24 were exactly the same as those described in the last phase of the training trials (recurrent face vs novel face). The other eight, the probe trials, consisted in presenting the same photograph of their keeper’s face opposite a novel face. These probe trials were interspersed between the training trials in a semi-random fashion (never more than twice consecutively and balancing presentation of the keeper’s face between the left and right side of the screen). During the probe trials, rewards were given for both images (keeper’s and novel face), the aim being to determine the horse’s spontaneous choice without creating a learning bias to touch the keeper’s rather than the novel face. Two types of probe test were conducted in two different sessions: one in which the same photograph of the current keeper’s face was presented eight times and the other involving the same photograph of their previous keeper’s face also presented eight times.

The current keeper had worked for 10 months in the stables where the horses were kept. The previous keeper had worked in the stables for five months, but had stopped working there six months before the experiment began and the horses never saw that person during that six-month interval. The previous and the current keeper were the same two people for all 11 horses. In each case, the keepers looked after the horses five days a week to let them out to pasture or to carry out simple activities with them to familiarize them with humans (training in hand mainly based on positive reinforcement).

### Control tests

A control test was conducted to check that the horses’ recognition of the keeper was based on the familiarity of the person in real life rather than on other clues (such as the fact that the same image reappeared eight times). The procedure was identical to that of the probe test with the keepers’ faces (a reward was given for both images), but this time it was the face of an unfamiliar person that was presented eight times, interspersed between 24 training trials. This control was conducted at the end of the experiment because we thought that it could degrade the horses’ responses consequently influencing the following tests.

### Preparation of the images

The images of the recurrent and control faces were digital photographs taken with a NIKON D3300. The photographs of novel objects and novel faces were obtained from the Internet. The faces were all of adult women. There was no similarities between the four recurrent and the keeper that could have been perceived as cues by the horses (eg. eye or hair color or style). All the images were edited using ImageJ software. The images were cropped and the background was white. Brightness and contrast were automatically adjusted to control for differences in lighting conditions. Life-size images (photographs and black circle) displayed on the screen were: 25 cm high, 20 + 2 cm wide.

### Data collection and analysis

Data were analyzed with XLSTAT software. The data from the pre-training and training stages were analyzed to determine whether animal performance increased over the sessions. For the pre-training sessions, all the horses carried out four sessions, and we compared the percentage of success (number of correct responses out of the 32 trials) for all the sessions using a Friedman test. For the different training stages, each horse carried out at least two sessions, but subsequently could move on to the following stage if it had reached the success criterion (75% of correct responses over two consecutive days). Thus, for the statistical analyses of all the horses (n = 11), we only compared performances between the first and the second session using a Wilcoxon test.

The data from the two probe tests and the control test were analyzed using Student t-tests to compare the overall performance of the 11 horses (number of correct responses out of 8 trials) compared to the chance level (50%). A t-test was also used to check for side bias. The threshold for statistical significance was set at P ≤ 0.05. We also calculated Cohen’s d = (mean 2 − mean 1)⁄s.d._pooled_ as a measure of effect size. Cohen’s d = 0.2 is considered a ‘small’ effect size, 0.5 represents a ‘medium’ effect size and 0.8 a ‘large’ effect size.

## Supplementary information

Supplementary Information.
