# Supplementary material for: Female horses spontaneously identify a photograph of their keeper, last seen six months previously
Source: Sci Rep. 2020 Apr 14;10:6302. doi: 10.1038/s41598-020-62940-w (PMC7156667; doi:10.1038/s41598-020-62940-w)
Supplement: Supplementary file 1 — Supplementary Information. [file 41598_2020_62940_MOESM1_ESM.pdf]

Female horses spontaneously identify a photograph of their keeper, last seen six months previously

Léa Lansade[a*], Céline Parias[a], Miléna Trösch[a], Fabrice Reigner[b], Violaine Colson[c], Ludovic Calandreau[a]

*Corresponding author

mail: lea.lansade@inrae.fr

phone number: 0033 247 427 279

Supplementary material

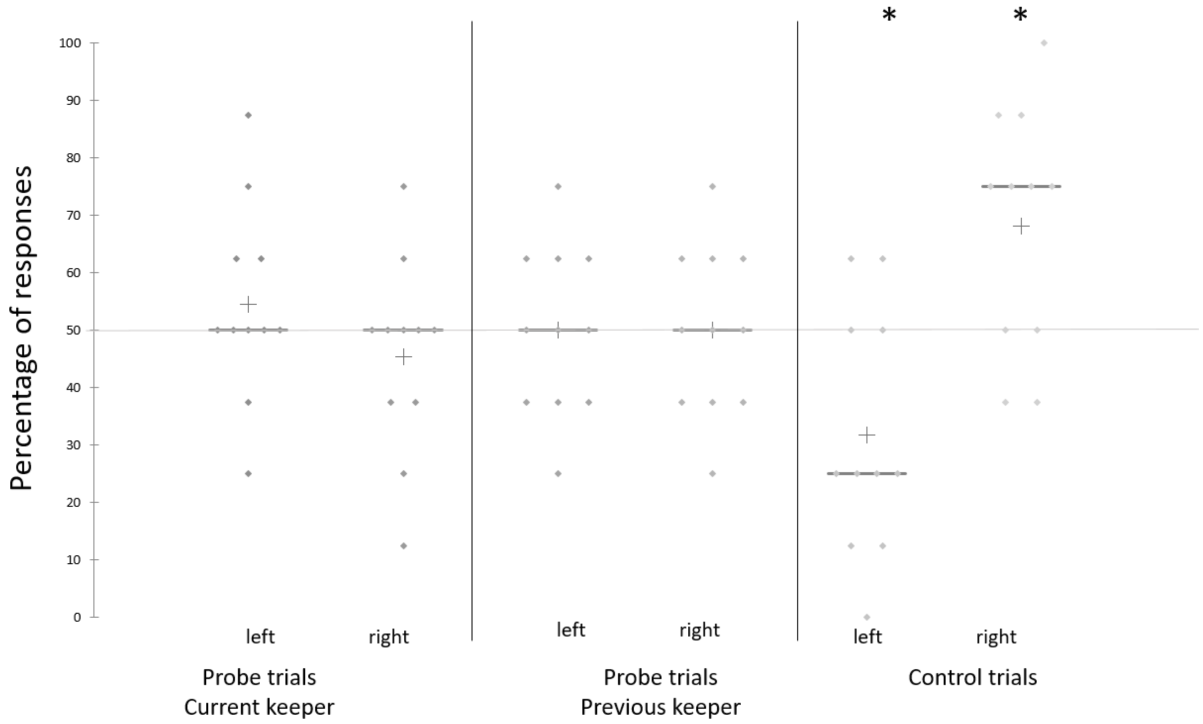

Supplementary Figure S1. Percentage of responses on the right or left side of the screen during the probe trials with the current or previous keeper and during the control trials (percentage calculated for four trials on the left and four trials on the right side of the screen per session)

** : P<0.05 ; t-test, calculated according to the level of chance (50%), N=11*

+: mean, ____: median
